# The first detection of influenza in the Finnish pig population: a retrospective study

**DOI:** 10.1186/1751-0147-55-69

**Published:** 2013-09-18

**Authors:** Tiina Nokireki, Taina Laine, Laura London, Niina Ikonen, Anita Huovilainen

**Affiliations:** 1 Finnish Food Safety Authority Evira, Mustialankatu 3, FI-00790 Helsinki, Finland; 2 National Institute for Health and Welfare, Mannerheimintie 166, FI-00270 Helsinki, Finland

**Keywords:** Porcine, Influenza A, H1N1, Serology, Phylogenetic analysis, Finland

## Abstract

**Background:**

Swine influenza is an infectious acute respiratory disease of pigs caused by influenza A virus. We investigated the time of entry of swine influenza into the Finnish pig population. We also describe the molecular detection of two types of influenza A (H1N1) viruses in porcine samples submitted in 2009 and 2010.

This retrospective study was based on three categories of samples: blood samples collected for disease monitoring from pigs at major slaughterhouses from 2007 to 2009; blood samples from pigs in farms with a special health status taken in 2008 and 2009; and diagnostic blood samples from pigs in farms with clinical signs of respiratory disease in 2008 and 2009.

The blood samples were tested for influenza A antibodies with an antibody ELISA. Positive samples were further analyzed for H1N1, H3N2, and H1N2 antibodies with a hemagglutination inhibition test.

Diagnostic samples for virus detection were subjected to influenza A M-gene-specific real-time RT-PCR and to pandemic influenza A H1N1-specific real-time RT-PCR. Positive samples were further analyzed with RT-PCRs designed for this purpose, and the PCR products were sequenced and sequences analyzed phylogenetically.

**Results:**

In the blood samples from pigs in special health class farms producing replacement animals and in diagnostic blood samples, the first serologically positive samples originated from the period July–August 2008. In samples collected for disease monitoring, < 0.1%, 0% and 16% were positive for antibodies against influenza A H1N1 in the HI test in 2007, 2008, and 2009, respectively.

Swine influenza A virus of avian-like H1N1 was first detected in diagnostic samples in February 2009. In 2009 and 2010, the avian-like H1N1 virus was detected on 12 and two farms, respectively. The pandemic H1N1 virus (A(H1N1)pdm09) was detected on one pig farm in 2009 and on two farms in 2010.

**Conclusions:**

Based on our study, swine influenza of avian-like H1N1 virus was introduced into the Finnish pig population in 2008 and A(H1N1)pdm09 virus in 2009. The source of avian-like H1N1 infection could not be determined. Cases of pandemic H1N1 in pigs coincided with the period when the A(H1N1)pdm09 virus was spread in humans in Finland.

## Background

Three influenza A virus subtypes, H1N1, H3N2 and H1N2, are circulating in the swine population in Europe [1]. The first outbreak of influenza in pigs was described in the UK before the 1950s [2]. The avian-like H1N1 was introduced into the swine population after the transmission of H1N1 virus from wild ducks to pigs in the late 1970s [3] and since 1979 it has replaced the antigenically and genetically distinguishable North American classical swine H1N1 viruses [2]. The H3N2 subtype of human origin formed a stable lineage in European pigs in the early 1970s, but reassortant H3N2 has been dominant since the mid-1980s [4]. The H1N2 subtype was first isolated in Great Britain in 1994 [5].

In the Nordic countries, H1N1 swine influenza was first reported in Denmark in 1981 [6] and in Sweden in the spring of 1983 [7]. Norway was free of swine influenza until the pandemic H1N1 influenza virus (A(H1N1)pdm09) was detected on Norwegian pig farms in 2009 at the same time as the virus spread in the human population [8]. In Finland, antibodies against influenza A virus (H1N1) as well as the avian-like influenza A (H1N1) virus were detected in porcine samples in February 2009.

In earlier studies in Finland, antibodies indicating the circulation of swine influenza have not been detected. In 1993, a swine influenza survey was carried out and blood samples from slaughtered pigs were examined. Antibodies against H1N1 were not found [9]. Boars were serologically screened for swine influenza antibodies before they entered boar stations from 1994 [10] until 2009 [11]. During 1997–2007, blood samples from pigs that originated from breeding herds and that were slaughtered from the test stations were tested for H1N1 antibodies [12].

Here, we describe a retrospective serological study that examined the time of entry of H1N1 swine influenza virus into the Finnish pig population. For the first time, we also report the detection of two types of H1N1 influenza A virus in pigs in Finland. The first vaccines against swine influenza were released for marketing at the beginning of 2010, which made this retrospective study possible.

## Methods

### Blood samples

#### Samples collected for disease monitoring

Each year between February and May, blood samples are randomly collected from sows for disease surveillance at all major swine slaughterhouses in Finland. The number of samples tested annually is presented in Table 1.

**Table 1:** Results from the analysis of blood samples collected for surveillance purposes from sows in slaughterhouses and tested using the hemagglutination inhibition test

***Year***	***Number of animals tested***	***H1N1 positive animals (%)***	***H1N2 positive animals (%)***	***H3N2 positive animals (%)***
2007	1192	1 (0.08)	14 (1.2)	11 (0.9)
2008	938	0 (0)	16 (1.7)	31 (2.6)
2009	1101	171 (15.5)	3 (0.3)	11 (1)

Samples from pigs in special health class farms producing replacement animals were also tested. In 2008 and 2009, a total of 472 samples from 42 farms and 465 samples from 31 farms, respectively, were analyzed for the presence of swine influenza antibodies.

#### Diagnostic samples

A total of 276 diagnostic blood samples taken from 18 farms in 2008 and 285 blood samples taken from 24 farms in 2009 that had reported clinical signs of respiratory disease were analyzed for the presence of swine influenza antibodies.

### Serological tests

Serological analyses of the blood samples were first carried out with influenza A antibody ELISA (ID Screen® Influenza A Antibody Competition, IdVet, France) according to the instructions of the kit manufacturer. ELISA-positivesamples were further analyzed using a hemagglutination inhibition (HI) test according to the operating procedure of the European Surveillance of Influenza in Swine with the antigens H1N1 (SW/Best/96), H1N2 (SW/Gent/7625/99) and H3N2 (SW/St. Oedenrode/96). All the antigens were provided by GD Animal Health Service, Deventer, NL.

### Virus detection and molecular studies

Diagnostic samples (dead pigs, organ samples, nasal swabs) submitted for laboratory examination from farms affected by an acute respiratory disease were used for virus detection when swine influenza was suspected by the veterinarian in the laboratory or on the farm.

Viral RNA was extracted from lung and swab suspensions or allantoic fluids of infected embryonated hen’s eggs by using a QiaAmp ViralRNA Mini Kit (Qiagen, Hilden, Germany). All samples used for virus detection were subjected to influenza A M-gene-specific real-time RT-PCR [13] and to A(H1N1)pdm09 -specific real-time RT-PCR [14]. Samples positive in real-time RT-PCR were inoculated into embryonated hen’s eggs according to the EU diagnostic manual for avian influenza in order to isolate the virus.

The complete coding regions of both the HA and NA genes of the strain A/Swine/Finland/si723/09 were amplified in three overlapping parts by using a OneStep RT-PCR Kit (Qiagen, Hilden, Germany). The three primer pairs for HA gene amplification were: 5′agcaaaagcaggggataattaaatc3′ and 5′tgatacaactgaaacatatgtatg3′, 5′caccaagtcatacacaaacaac3′ and 5′ggcattatatgtccatacatc3′, and 5′aaatgagcagggatctggttac3′ and 5′gcacactttgaaatccaagtc3′. For NA gene amplification, the primer pairs were: na1 [15] and 5′actgactcaaatcttgagttg3′, 5′ccytgctraatgacaaacattc3′ and 5′tactggaccacaactgcctg3′, and 5′tcaaataggatacatatgcagtg3′ and 5′ttatatggtctcgtattagtag3′. In all PCRs the reaction volume was 50 μl and the temperature profile of cDNA synthesis and amplification was: 50°C for 30 min, 95°C for 15 min and 40 cycles of 95°C for 30 s, 58°C for 30 s, 72°C for 1 min. In addition, the middle part of both genes of all Finnish isolates (only the HA gene of si522/09) detected in 2009–2010 was amplified and sequenced in the same manner to confirm having the same H1N1 subtype as the si723/09 strain.

The sequencing was carried out using a BigDye Terminator Cycle sequencing kit v3.1 and an ABI3100 Avant automatic sequencer, and the primers used in the PCRs.

The complete HA and NA genes of pandemic strains A/Swine/Finland/si5688/09, A/Swine/Finland/si3431/10, and A/Swine/Finland/si4171/10 were amplified and sequenced as previously published [16].

**Figure 1:**
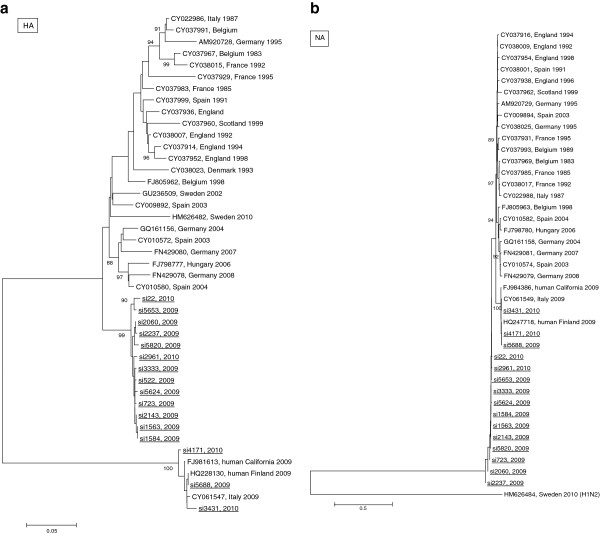
**Phylogenetic relationships of the Finnish influenza A viruses isolated in swine in 2009-2010 [**[16]**,**[20]**-**[25]**]. (a)** Phylogenetic tree based on 743 nucleotides of haemagglutinin gene. **(b)** Phylogenetic tree based on 560 nucleotides of neuraminidase gene.

The sequences were edited and the nucleotide identities calculated with the EMBOSS package [17]. The phylogenetic analyses were carried out with the middle part sequences of HA and NA genes. The 744 nucleotides of HA and 560 nucleotides of NA genes of the Finnish strains and over 20 swine influenza A (H1N1) and three A(H1N1)pdm09 strains originating in swine and in humans were aligned with the program ClustalW [18], and neighbor-joining phylogenetic trees (Figure 1a and 1b) were created with the program MEGA 5.05 [19]. The data were bootstrapped 1000 times, and only values higher than 85% are shown. One strain per farm was included in the analysis.

## Results

### Serology

#### Samples collected for disease monitoring

HI test results of the samples collected for disease monitoring are presented in Table 1. The only positive H1N1 sample (<0.1% of samples tested) in 2007 had a titer of 1:16. In 2008, no seropositive samples were detected. However, in 2009, 15.5% of the surveillance samples were H1N1 positive in the HI test. These positive samples from 2009 originated from 120 farms, which represented 29.9% of the farms tested. Antibodies against H1N2 and H3N2 were detected in a few samples.

#### Samples from special health class farms producing replacement animals

HI test results of the samples from special health class farms producing replacement animals show that in 2008 one farm out of 42 (2,3%) and in 2009 four out of 31 (12,9%) farms was serologically H1N1 positive. The first H1N1-positive serum sample of a special health class farm was taken in July 2008. No H1N2 or H3N2 seropositive farms were detected.

#### Diagnostic samples

**Figure 2:**
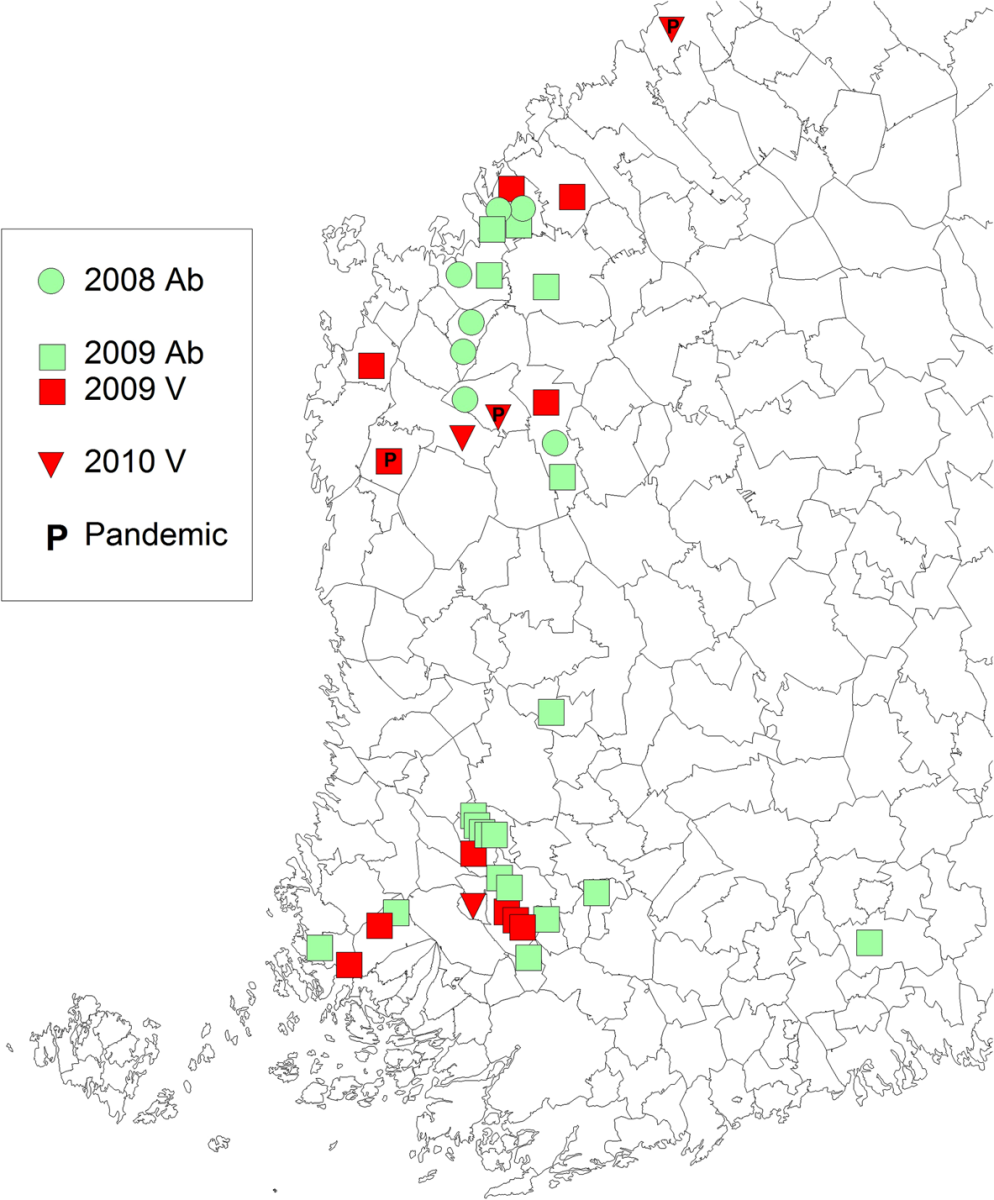
Map indicating the locations of farms where either antibodies (Ab) against swine influenza were detected or avian-like swine influenza H1N1 (V) or A(H1N1) pdm09 (P) viruses were isolated during 2008 to 2010.

The results of samples taken from pigs in farms with clinical signs of acute respiratory disease show that in 2008 seven out of 18 (38,8%) farms tested had H1N1 antibodies. None of the farms had H1N2 or H3N2 antibodies. The first H1N1-seropositive samples were taken in August 2008. All 7 farms where H1N1 antibodies were detected in 2008 were located in 5 adjoining municipalities in Ostrobothnia (Figure 2). In 2009, a total of 24 farms submitted serological samples for antibody testing due to acute respiratory signs. Of these, samples from 13 (54,2%) farms tested positive for H1N1 antibodies. All antibody- or virus-positive farms with respiratory signs were located either in the same area as the positive farms in 2008 or in a separate area in southwest Finland (Figure 2).

### Virus detection

In February 2009, the avian-like swine H1N1 influenza A virus (si522/09) was detected for the first time in porcine lung samples from a Finnish farm experiencing respiratory disease in sows and weaned pigs. Soon after that, a similar influenza A virus was detected on two other pig farms (si723/09 and si1563/09) with acute respiratory disease. These farms were localized in the same area. In 2009 and 2010 this swine H1N1 influenza virus was detected on 12 farms and two farms, respectively.

The A(H1N1)pdm09 virus was diagnosed in pigs on one farm in November 2009 and on two farms in 2010 (one in August and one in November). No influenza viruses have been detected in porcine samples in 2011 and 2012. The locations of the infected farms are indicated in Figure 2 and the types of diagnostic samples related to the detection of H1N1 influenza virus are presented in Table 2.

**Table 2:** The types of diagnostic porcine samples in which influenza A virus was detected

***Sample type***	***Number of positive farms***	
	***Avian-like H1N1 virus***	***Pandemic H1N1 virus A(H1N1) pdm09***
Year 2009 Lung tissue	7	0
Nasal swabs	5	1
Year 2010*Lung tissue	1	1
Nasal swabs	1	2

*On one farm, A(H1N1)pdm09 virus was detected in both sample types.

### Phylogenetic studies

The coding regions of HA and NA genes of si723/09 were 1701 and 1410 nucleotides long, respectively. The 744-nt-long HA and 560-long-NA gene fragments of these Finnish swine influenza strains differed from each other at maximum by 1.7% and 1.3%, respectively. The strains from 13 farms grouped together and shared an approximately 94% nucleotide identity in both HA and NA genes with the European swine influenza A (H1N1) viruses of avian origin (Figure 1).

The three pandemic strains, A/Swine/Finland/si5688/09, A/Swine/Finland/si3431/10, and A/Swine/Finland/si4168/10, differed from each other at maximum by 0.9% in HA and 0.8% in NA. They clustered together with the Finnish human pandemic strains [16] and the A/California/2009 strain [24], sharing an approximately 99.2% nucleotide identity in the HA gene and 99.4% nucleotide identity in the NA gene. The pandemic HA gene cluster clearly differed from the swine influenza A (H1N1) avian origin strains (about 74% nt identity) (Figure 1a), whereas the NA gene clustered together with both classical swine influenza H1N1 viruses and pandemic H1N1 viruses, as expected (Figure 1b).

The GenBank accession numbers for strains sequenced in this study are: complete HA and NA sequences for si723/2009, KC261841 and KC261842, respectively. Complete HA and NA sequences of pandemic strains si5688/2009, si3431/2010 and si4171/2010: KC336409–KC336411 and KC336412–KC336414. Middle part HA sequences of other strains: KC261843 and KC293799–293809, and middle part NA sequences of other strains: KC292810–293820.

## Discussion

Here, we describe the entry of two swine influenza A (H1N1) viruses into the Finnish pig population. Based on a retrospective serological study, the porcine European avian-like H1N1 virus was introduced in 2008. However, the virus was first detected in February 2009. The A(H1N1)pdm09 virus was detected in Finnish pigs for the first time in November 2009.

Before 2008, Finland was considered to be free from swine influenza. According to the results from the samples collected for disease monitoring in 2007, 2008, and 2009, respectively, <0.1%, 0%, and 15.5% of the blood samples were serologically positive for antibodies against H1N1 in the HI test. The only positive H1N1 sample in 2007 had a titer of 1:16 and is not considered to be a real indication of H1N1 infection in Finland.

The numbers of H3N2 and H1N2 seropositive samples in 2007 and 2008 were low. In 2005–2007, seasonal influenza in humans in Finland was mainly caused by H3N2 influenza viruses [26]. Human H3N2 viruses have been recovered from pigs [1] and it is possible that H3N2 seropositive samples were due to infection with human H3N2 viruses and the low H1N2 titers could be explained by reactions to the N2 component. The number of H1N2 and H3N2 positive samples did not increase over time. Therefore they cannot be considered as indication of a true circulation of these viruses in the Finnish pig population.

The source of avian-like H1N1 influenza virus infection in Finnish pigs remained unresolved. From 1995 until 2010, live production pigs were only imported from Norway [27]. Norway was free from swine influenza until 2009, when A(H1N1)pdm09 was diagnosed on Norwegian pigs at the same time as in humans [8]. The restrictive policies in Finland concerning the import of live pigs are a result of a voluntary system supported by the industry to preserve the health status of Finnish pigs [27]. Concerning the swine H1N1 virus detected in Finland, the geographically closest swine influenza H1N1 strains have been isolated in Sweden in 2002 [25], and H1N1 continues to circulate in the Swedish swine population [28]. Strains belonging to the same phylogenetic virus group have been isolated in many European countries since the 1980s, indicating the wide circulation of these types of viruses (Figure 1).

The A(H1N1)pdm09 strains detected in swine and humans were similar, with si5688/09 and HQ228130 human strains even sharing 100% nucleotide identity. However, no epidemiological data are available and the direction of virus transmission between humans and swine is impossible to estimate. As in the study on the Finnish human A(H1N1)pdm09 strains [16], no changes possibly leading to an increased epidemic potential or exceptionally high virulence of viruses were seen.

A new respiratory disease like swine influenza was expected to be noticed based on clinical signs due to the good health status of the Finnish pigs. Finland is free from Aujeszky’s disease [29,30]. Porcine reproductive and respiratory syndrome (PRRS) has never been detected in Finland [31]. Due to additional guarantees for transmissible gastroenteritis (TGE) [30], blood samples are annually monitored for TGE antibodies and cross-reactive porcine respiratory coronavirus (PRCV) antibodies, indicating PRCV infection has never been found in Finnish pigs. In addition, freedom from *Mycoplasma hyopneumoniae* has been one of the basic criteria for the national health program for elite breeding herds that started in 1983 [32], voluntary health classification programs for conventional pig herds launched by several slaughterhouse co-operatives in the 1990s [33,34], and the current health classification of pig herds [35]. In 2007, very few sow farms were either *M. hyopneumoniae* positive or had an unknown status concerning *M. hyopneumoniae*[36].

Some pig farmers reported short-lasting respiratory signs in pigs in the fall of 2008, when Finland was still considered to be free from swine influenza. Due to the emphasized importance of *M. hyopneumoniae* in the health classification system and knowledge about the restrictive policy concerning imports of live pigs, sampling and testing at that time were mainly conducted to rule out *M. hyopneumoniae* infection. Retrospectively, a higher number of H1N1 seropositive samples were found in pigs in herds with clinical signs than in samples of pigs collected for disease monitoring and in samples of pigs in special health class farms. Avian-like swine H1N1 does not necessarily cause coughing in pigs that are free from *M. hyopneumoniae* infection [37], and subclinical infections are possible [38,39]. This could have contributed to the delayed detection of influenza virus in Finland. Virus is expected to be found in the respiratory tract of pigs only during the first week after infection, and paired serum samples are recommended for serology that include a second sampling 3 to 4 weeks after acute disease [1]. Mild and short-lasting clinical signs are also reported in pigs infected with A(H1N1)pdm09 virus [40]. Mild and short-lasting clinical signs may not motivate extensive sampling.

## Conclusions

Based on our study, the avian-like swine H1N1 influenza virus was introduced into the Finnish pig population in 2008 and A(H1N1)pdm09 virus in 2009. The source of the avian-like swine H1N1 virus infections remains unresolved. The mild signs of avian-like swine H1N1 influenza can allow a wide spread of the introduced virus in naïve pig populations before detection especially, when pigs are free from significant respiratory pathogens like Mycoplasma hyopneumoniae. The A(H1N1)pdm09 virus was detected in the time period when pandemic H1N1 was spreading in the human population in Finland.

## Competing interests

The authors declare that they have no competing interests.

## Authors’ contributions

TN participated in the diagnostics of samples submitted for serology and virus detection. She participated in analyzing the data and in drafting the manuscript. TL participated in the diagnostics of samples submitted to the laboratory for pathological examination. She participated in analyzing the data and in drafting the manuscript. LL participated in analyzing the data and in drafting the manuscript. NI sequenced the pandemic H1N1 strains and participated in analyzing the data and in drafting the manuscript. AH designed and participated in the molecular diagnosis, sequenced the swine influenza strains, performed the phylogenetic analyses and participated in drafting the manuscript. All authors read and approved the final manuscript.
